# Magnetic Ion Imprinted Polymers (MIIPs) for Selective Extraction and Preconcentration of Sb(III) from Environmental Matrices

**DOI:** 10.3390/polym14010021

**Published:** 2021-12-22

**Authors:** Silindokuhle Jakavula, Nkositetile Raphael Biata, Kgogobi M. Dimpe, Vusumzi Emmanuel Pakade, Philiswa Nosizo Nomngongo

**Affiliations:** 1 Department of Chemical Sciences, Doornfontein Campus, University of Johannesburg, Doornfontein 2028, South Africa; jakavulasilindokuhle@yahoo.com (S.J.); raphaelbiata@gmail.com (N.R.B.); mdimpe@uj.ac.za (K.M.D.); 2 Department of Science and Innovation-National Research Foundation South African Research Chair Initiative (DSI-NRF SARChI), Nanotechnology for Water, University of Johannesburg, Doornfontein 2028, South Africa; 3 Department of Chemistry, Vaal University of Technology, Private Bag X 021, Vanderbijlpark 1911, South Africa; vusumzip@vut.ac.za

**Keywords:** ion imprinted polymers, antimony(III), magnetic solid-phase extraction, environment matrices, Sb(III) IIP@Fe_3_O_4_@CNF@SiO_2_

## Abstract

Antimony(III) is a rare element whose chemical and toxicological properties bear a resemblance to those of arsenic. As a result, the presence of Sb(III) in water might have adverse effects on human health and aquatic life. However, Sb(III) exists at very ultra-trace levels which may be difficult for direct quantification. Therefore, there is a need to develop efficient and reliable selective extraction and preconcentration of Sb(III) in water systems. Herein, a selective extraction and preconcentration of trace Sb(III) from environmental samples was achieved using ultrasound assisted magnetic solid-phase extraction (UA-MSPE) based on magnetic Sb(III) ion imprinted polymer-Fe_3_O_4_@SiO_2_@CNFs nanocomposite as an adsorbent. The amount of antimony in samples was determined using inductively coupled plasma optical emission spectrometry (ICP-OES). The UA-MSPE conditions were investigated using fractional factorial design and response surface methodology based on central composite design. The Sb(III)-IIP sorbent displayed excellent selectivity towards Sb(III) as compared to NIIP adsorbent. Under optimised conditions, the enrichment factor, limit of detection (LOD) and limit of quantification (LOQ) of UA-MSPE/ICP-OES for Sb(III) were 71.3, 0.13 µg L^−1^ and 0.44 µg L^−1^, respectively. The intra-day and inter-day precision expressed as relative standard deviations (%RSDs, n = 10 and n = 5) were 2.4 and 4.7, respectively. The proposed analytical method was applied in the determination of trace Sb(III) in environmental samples. Furthermore, the accuracy of the method was evaluated using spiked recovery experiments and the percentage recoveries ranged from 95–98.3%.

## 1. Introduction

Antimony (Sb) is a toxic metalloid that exists at very ultra-trace levels in the environment [1,2,3]. Inorganic compounds of Sb are said to be more toxic than other metal compounds [4]. Human beings may be exposed to Sb by breathing air, drinking water and eating food that is contaminated with Sb [5]. The toxicity of Sb depends on its chemical species. For instance, Sb(III) is ten times more toxic than Sb (V) [6] and Sb(III) shows high affinity towards red blood cells [5]. Antimony levels in the environment have been elevated due to its rapid use in batteries, plastics, paints, alloys and semiconductors [7]. In addition, Sb is used as a catalyst in the production of poly(ethylene terephthalate) (PET), which is used in plastic bottles [8]. These PET bottles are used as packages for beverages and water [9,10]. As such, Sb is often detected in bottled water and beverages because of leakage from PET through storage [11,12].

Due to the toxicity of Sb, there are numerous analytical techniques that have been used for the determination of total and Sb species. These include inductively coupled plasma mass spectrometry (ICP-MS) [13], graphite furnace atomic absorption spectrometry (GF-AAS) [14] and inductively coupled plasma optical emission spectrometry (ICP-OES) [15], etc. Due to low concentrations of Sb and complexity of sample matrices, sample preparation is required prior to its determination [16]. Several researchers have developed various sample preparation methods, which include solid phase extraction (SPE) [2], ultrasound-assisted cloud point extraction (UA-CPE) [17], dispersive micro-solid phase extraction (DSPME) [18] and magnetic solid phase extraction (MSPE) [15], among others.

Among these methods, solid phase-based procedures have attracted a lot of attention. This is due to their attractive features such as flexibility, simplicity and choice of adsorbent. Therefore, in order to successfully and selectively extract Sb in various matrices, the choice of suitable adsorbent is required [19]. As a result, several sorbents have been used for extraction and preconcentration of Sb. These include Mg-Fe-OH- layered double hydroxide [20], zirconium oxide-carbon nanofibres [21], reduced graphene oxides/Mn_3_O_4_ [22], magnetic nickel ferrite (NiFe_2_O_4_) nanoparticles [23] and ion imprinted polymers (IIPs) [19], to name a few. Ion imprinted polymers are one of the promising sorbents that have been developed for selective extraction and preconcentration of trace metal ions [24,25].

Over the past years, IIPs have attracted attention for the determination, speciation and removal of metal ions due to their advantages, such as simplicity, high adsorption capacity, high selectivity, low costs, reusability and high extractions efficiency [26]. The general procedure for the synthesis of IIPs involves the formation of a metal ion (template) complex with a suitable ligand followed by copolymerisation in the presence of cross-linker, initiator and a monomer where a polymer matrix with recognition sites is formed [27]. Various researchers have reported they explored the use of IIPs as adsorbents for the removal and speciation of toxic trace metals, such as Hg(II) [28], Pb(II) [29], Co(II) [30], Pb(II) [31], As(II) [32], Sb(III) [19,26] and Cd(II) [33], among others. In addition, there are also reports on the use of novel molecular imprinted electrochemical sensor for the detection of different pollutants [34,35,36,37,38]. These studies have shown that the combination of molecularly imprinted polymer (MIP) or IIP with other nanomaterials, such as carbon materials, has attracted great consideration due to the synergetic effect which enables a high mass transfer rate [34,35,36,38]. In addition, these nanomaterials serve as excellent substrate in surface imprinting processes and some of these materials (e.g., magnetic nanoparticles) provide better separability and reusability. According to the literature search, when compared to other toxic trace metals, few studies have explored the use of IIPs for selective extraction, preconcentration or removal of Sb [6,19,27,39,40,41,42]. Notwithstanding the fact that there are already existing reports on the application of IIPs for the analysis of Sb in water, there is a need for continuously monitoring the amount of Sb in these matrices.

Therefore, the aim of this study was to prepare Sb(III)-IIP by surface imprinting technique, using styrene as a monomer and Fe_3_O_4_@CNFs@SiO_2_ nanocomposite as a supporting substrate. Carbon nanofibres were chosen because of their attractive properties, such as large surface area, strong interaction with various substances as well as high affinity towards metals. However, the major drawback of using CNFs as support for IIPs is separability and regeneration. Therefore, anchoring of magnetic nanomaterials in CNFs matrix allows better separation and reusability. Lastly, incorporating mesoporous silica to magnetic CNFs leads to an excellent IIP carrier with large surface areas and tuneable pore sizes. The Sb(III)-IIP-Fe_3_O_4_@CNFs@SiO_2_ was characterised using X-ray diffraction (XRD), Fourier-transform infrared spectroscopy (FTIR), scanning electron microscopy (SEM), energy dispersive X-ray spectroscopy (EDS) and transmission electron microscopy (TEM). The prepared Sb(III)-IIP-Fe_3_O_4_@CNFs@SiO_2_ was used as adsorbent for selective extraction and preconcentration of Sb(III) in surface water samples prior to ICP-OES analysis. Factors affecting the preconcentration process were optimised using a multivariate approach.

## 2. Materials and Methods

### 2.1. Reagents and Materials

Analytical grade chemicals and ultrapure water were employed during the experiments. Antimony single standard (1000 mg L^−1^), ammonium pyrrolidine dithiocarbamate (APDC), iron (II) chloride tetrahydrate, carbon nanofibres (CNFs), hydrochloric acid, iron (III) chloride hexahydrate, ethanol, ammonia (25%), tetraethyl orthosilicate (TEOS), ethanol, 2-methoxy ethanol, antimony(III) chloride, styrene, ethylene glycol dimethacrylate (EGDMA), chloroform, 1,1′-azobisisobutyronitrile (AIBN) and nitric acid (65%) were obtained from Sigma-Aldrich (St. Louis, MO, USA).

### 2.2. Synthesis of Fe_3_O_4_ Coated with CNFs

The synthesis of Fe_3_O_4_ coated with CNFs was conducted according to previous studies [43]. To describe the method briefly, appropriate amounts of FeCl_2_ (2.50 g) and FeCl_3_ (6.82 g) were dissolved in deionised water and 2 mL of HCl was added in the mixture to facilitate the complete dissolution of the iron salts. About 2.5 g of CNFs were dispersed in the solution prepared above under continuous stirring. To precipitate the final product, about 250 mL of 1.5 mol L^−1^ NH_3_ was added (dropwise) to the mixture until the pH of the solution ranged from 11–12. The mixture was placed on a heater stirrer and the temperature was set at 80–90 °C. The mixture was stirred until the resultant black product has formed. The mixture was cooled to ambient temperature and the Fe_3_O_4_@CNFs nanocomposite was collected using external magnet. The Fe_3_O_4_@CNFs nanocomposite was washed with ethanol–water (50:50) mixture and dried at 100 °C for 2 h.

### 2.3. Synthesis of Fe_3_O_4_@CNFs@SiO_2_ and Sb(III)-IIP-Fe_3_O_4_@SiO_2_@CNFs Nanocomposites

Synthesis of the Fe_3_O_4_@CNFs@SiO_2_ nanocomposite was carried according to previous studies with minor adjustments [33]. Firstly, Fe_3_O_4_@CNFs (2.0 g) was dispersed in alcoholic aqueous solution (45 mL of H_2_O and 100 mL of ethanol). The mixture was heated at 70 °C and 4.5 mL of ammonium solution (25 wt. %) was added while stirring. The mixture was further stirred continuously for 15 min. Ethanolic solution (50 mL) containing TEOS (8.25% *v*/*v*) was added dropwise for 90 min and the mixture was stirred at 70 °C continuously for 6 h. The final product was separated from the supernatant using external magnet. The Fe_3_O_4_@CNFs@SiO_2_ nanocomposite was subsequently washed with ethanol (to remove unreacted TEOS) followed by rinsing with distilled water and dried at 60 °C in an oven for 12 h. The synthesis of Sb(III)-IIP was conducted according to [44] and the detailed procedure is presented in Supplementary Information.

### 2.4. Ultrasonic-Assisted Magnetic Solid Phase Extraction (UA-MSPE) Procedure

The UA-MSPE method was performed according to [45]. The extraction procedure was conducted as follows: 10–100 mg of Sb(III)-IIP-Fe_3_O_4_@SiO_2_@CNFs adsorbent was placed in a centrifuge tube followed by the addition of 10 mL of sample solution at pH = 2, pH = 5.5, pH = 9. The extraction and preconcentration steps were achieved by the dispersion of the adsorbent in the sample via ultrasonication for 5–30 min. The adsorbent containing the adsorbed analyte was separated from the aqueous solution by application of an external magnet at the base of the centrifuge tube. The supernatant was filtered using 0.22 µm PVDF membrane and the filtrate was analysed using inductively coupled plasma-optical emission spectrometer (ICP-OES) (iCAP 6500 Duo, Thermo Scientific, Hemel Hempstead, UK). The ICP-OES conditions are stated in Table S1.

### 2.5. Optimisation Strategy

The 2^6−2^ fractional factorial design (FrFD) was used for the screening of the most influential experimental factors affecting the extraction and preconcentration procedure. These include elution time (ET), sample pH, eluent volume (EV), eluent concentration (EC), sonication time (ST) and mass of adsorbent (MA). The independent and their levels are shown in Table 1.

After the screening process using FrFD, the most influential parameters were found to be MA and pH. These factors were optimised using response surface methodology (RSM) based on central composite design (CCD).

### 2.6. Adsorption Experiments

The adsorption equilibrium experiments were carried out under optimised conditions. Briefly, 56 mg of Sb(III)-IIP-Fe_3_O_4_@SiO_2_@CNFs and IIP-Fe_3_O_4_@SiO_2_@CNFs were added into 30.0 mL of a synthetic sample that contains Sb(III) at initial concentrations ranging from 2–10 mg/L. The samples were sonicated for 10 min at 25 °C and followed by external magnet separation. The supernatant was filtered and analysed for residual Sb(III) using ICP-OES. The amount of Sb(III) in the synthetic samples before adsorption and procedure blanks were determined using ICP-OES. The analytical results obtained were processed using Equation (1) to estimate adsorption capacity (*q_e_*, mg/g).

(1) $$q_{e}=\frac{\left(C_{0}-C_{e}\right)V}{m}$$

where *C*_0_ and *C_e_* are initial and equilibrium concentrations (mg/L) of Sb(III), *V* is the volume of the sample (L) and m is the mass of the adsorbent (g).

### 2.7. Selectivity Experiments

The selectivity studies were performed by placing 56 mg of Sb-IIP and NIP into 100 mL sample bottle containing 30 mL of the sample containing Sb(III), Al(III), Cd(II), Cu(II), Sn(IV) and Zn(II) at 10 mg/L (pH = 3). The samples were agitated using ultrasonic bath for 10 min at 25 °C. The remaining concentrations of Al(III), Cd(II), Cu(II), Sb(III), Sn(IV) and Zn(II) were determined using ICP-OES. Parameters such as distribution ratio (D), selectivity coefficient and relative selectivity coefficient were calculated according to Reference [20].

## 3. Results and Discussion

### 3.1. Characterisation

#### 3.1.1. X-ray Powder Diffraction (XRD)

**Figure 1:**
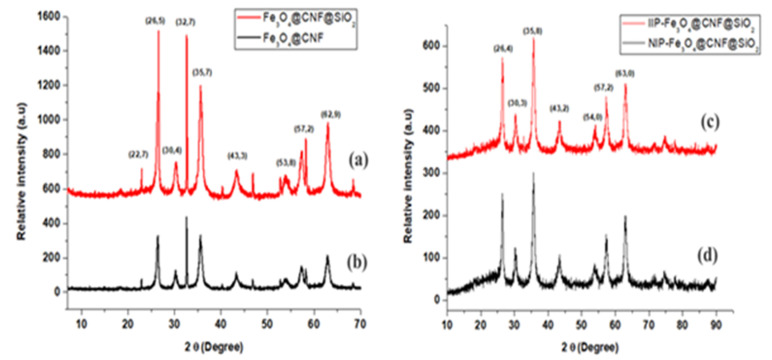
XRD patterns of (**a**) Fe_3_O_4_@CNFs, (**b**) Fe_3_O_4_@SiO_2_@CNFs, (**c**) IIP-Fe_3_O_4_@SiO_2_CNFs and (**d**) NIP-Fe_3_O_4_@SiO_2_@CNFs.

A PANalytical X’Pert Pro X-ray diffraction (XRD, PANalytical, Almelo, The Netherlands) was used to assess the crystalline structure of materials. Figure 1 displays the XRD patterns for Fe_3_O_4_@CNFs, Fe_3_O_4_@SiO_2_@CNF, Sb(III) IIP-Fe_3_O_4_@SiO_2_@CNFs and NIP-Fe_3_O_4_@SiO_2_@CNF. Figure 1a,b displays peaks at 2θ values of 26.5°, 30.4°, 32.7°, 43.3°, 53.8°, 57.2° and 62.9°, which are ascribed to Fe_3_O_4_ nanoparticle, confirming successful synthesis and incorporation of the magnetic nanoparticles. Figure 1c,d shows the diffractograms for Sb(III) IIP-Fe_3_O_4_@SiO_2_@CNFs and NIP-Fe_3_O_4_@SiO_2_@CNF and the peaks for the magnetic composite were observed at 2θ values of 26.4, 30.3°, 35.8°, 43.2°, 54.0°, 57.2° and 63.0° with reduced intensity. These results confirmed the loss of magnetic nanoparticles during the polymerisation process.

#### 3.1.2. Fourier-Transform Infrared Spectroscopy (FTIR)

**Figure 2:**
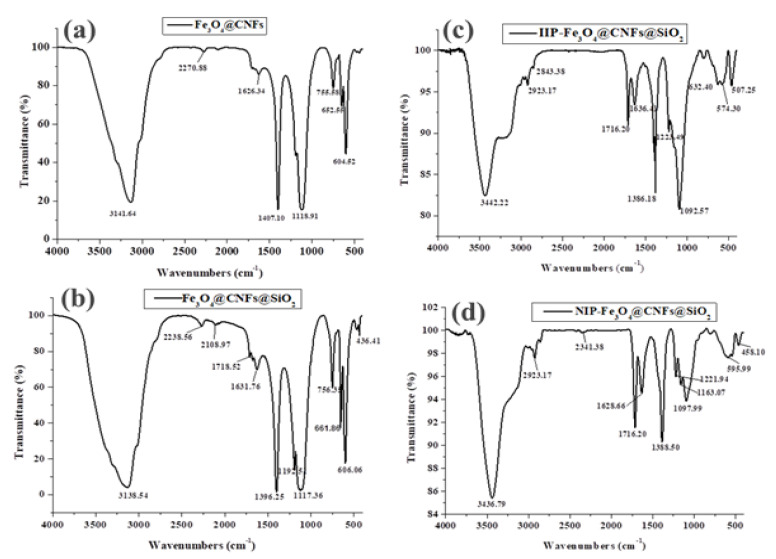
FTIR spectra of (**a**) Fe_3_O_4_@CNFs, (**b**) Fe_3_O_4_@SiO_2_@CNFs, (**c**) IIP-Fe_3_O_4_@@SiO_2_CNFs and (**d**) NIP-Fe_3_O_4_@SiO_2_@CNFs.

The structural properties of the prepared material were investigated using Perkin Elmer spectrum 100 Fourier-transform infrared spectrometer (FTIR, Waltham, MA, USA). (Figure 2). Figure 2a shows the spectrum of Fe_3_O_4_@CNFs and the peak at 604.5 cm^−1^ was assigned to the absorption of the Fe–O bond in Fe_3_O_4_. The peak at 1626 cm^−1^ was assigned to the C–C stretching associated with the nanofibre surface defects [46]. The peak at 3142 cm^−1^ was assigned to the –OH stretching of the CNFs. Figure 2b showed new peaks at 756.4 and 1117 cm^−1^, which were assigned to Si–O vibration and bending vibration of the Si–O–Si bond [47]. These findings confirmed the incorporation of SiO_2_ in nanocomposite matrix. Figure 2c,d exhibited characteristic bands of the polymeric matrix showing the presence of styrene–EGDMA polymers in the sample [48]. The peaks observed for the IIP-Fe_3_O_4_@SiO_2_@CNFs and NIP-Fe_3_O_4_@SiO_2_@CNFs were: 2923.17 cm^−1^ aliphatic C–H; 1716.20 cm^−1^ for C=O; and 1157.40 and 1153.89 cm^−1^ for C–O, which is assigned to the EDGMA ester group [48]. In Figure 2c, the C–S band of the APDC shifted from 595.99 (free APDC, Figure 2d) to 632.40 cm^−1^ in IIP, which indicated the formation of complex between Sb(III) and APDC [44].

#### 3.1.3. Scanning Electron Microscope/Energy Dispersive X-ray Spectroscopy (SEM/EDS)

**Figure 3:**
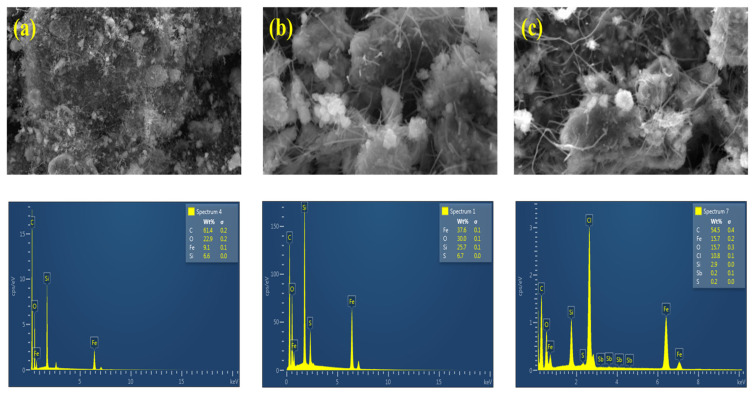
SEM and EDX images of (**a**) Fe_3_O_4_@SiO_2_@CNFs, (**b**) NIP-Fe_3_O_4_@SiO_2_@CNFs and (**c**) Sb(III) IIP-Fe_3_O_4_@SiO_2_@CNFs.

The morphological properties and elemental composition of the synthesised materials were investigated using scanning electron microscopy (SEM, TESCAN VEGA 3 XMU, LMH instrument, Tescan Company, Brno, Czech Republic)) coupled with energy dispersive X-ray spectroscopy (EDS). Figure 3 presents the SEM images and respective EDS spectra for (a) Fe_3_O_4_@SiO_2_@CNFs, (b) NIP-Fe_3_O_4_@SiO_2_@CNFs and (c) Sb(III) IIP-Fe_3_O_4_@SiO_2_@CNFs. The SEM/EDS was used to confirm the morphological elemental changes of the nanocomposites. Figure 3a confirmed the incorporation of magnetic nanoparticles on the surface of the carbon nanofibres. Moreover, the elemental analysis results of Fe_3_O_4_@SiO_2_@CNFs nanocomposite confirmed the presence of expected elements including C, O, Si and Fe in the ternary nanocomposite. Figure 3b,c reveals the growth of IIP on the surface of Fe_3_O_4_@SiO_2_@CNFs nanocomposite. In addition, the presence of C, Fe, O, Si and S in Figure 3b confirms that the surface imprinting was successful. Furthermore, the presence of Sb in Figure 3c confirm that the Sb(III) IIP-Fe_3_O_4_@SiO_2_@CNFs was successfully synthesised. The presence of Cl was from antimony chloride which was used during the synthesis of IIP.

#### 3.1.4. Transmission Electron Microscopy (TEM)

**Figure 4:**
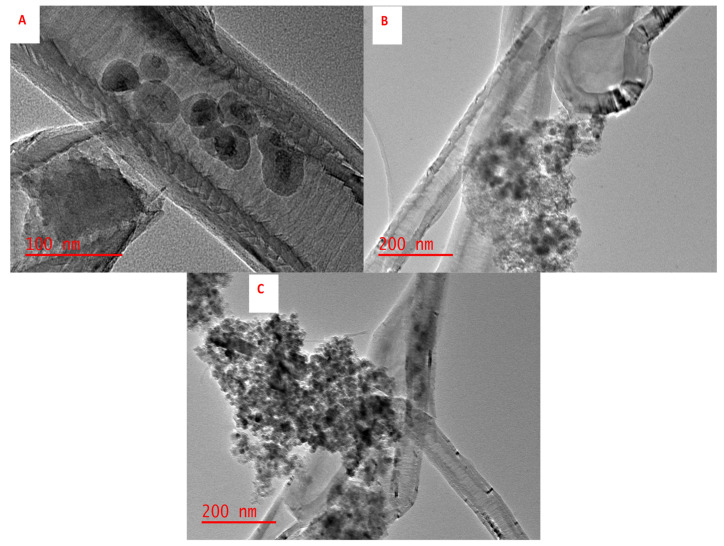
TEM images of (**A**) Fe_3_O_4_@SiO_2_@CNFs, (**B**) NIP-Fe_3_O_4_@SiO_2_@CNFs and (**C**) IIP-Fe_3_O_4_@SiO_2_@CNFs.

The transmission electron microscopy (TEM, JEM-2100, JEOL, Tokyo, Japan) was used to investigate the nano structure and particle size of the adsorbents. Figure 4 Illustrates the TEM images of (A) Fe_3_O_4_@SiO_2_@CNFs, (B) NIP-Fe_3_O_4_@SiO_2_@CNFs and (C) IIP-Fe_3_O_4_@SiO_2_@CNFs. Figure 4A reveals that spherical shape of Fe_3_O_4_@SiO_2_ nanocomposite evenly dispersed in the surface of carbon nanofibres. Figure 4B,C shows the growth of the polymer on the surface of Fe_3_O_4_@SiO_2_@CNFs nanocomposite.

### 3.2. Optimisation Strategy

**Figure 5:**
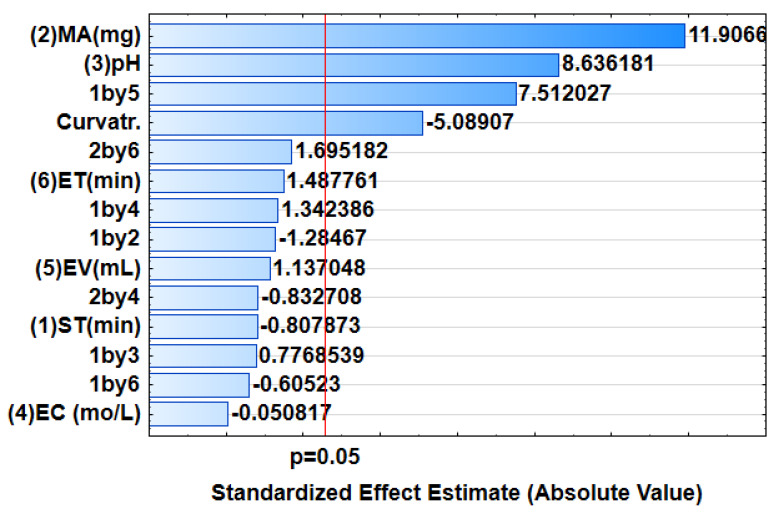
Pareto chart of the standardised effects for the extraction and preconcentration of Sb(III).

The fractional factorial design 2^6−2^ (FrFD) was used for selection of the most influential experimental parameters (eluent concentration (EC), sonication time (ST), elution time (ET), eluent volume (EV), mass of adsorbent (MA) and sample pH). The design matrix and the analytical response are presented in Table S2. The data was processed using Statistica version 13 software. The analysis of variance (ANOVA) was used to examine the significance of each independent factors. ANOVA results presented as Pareto chart (Figure 5) was used to assess the importance of independent variables and their interactions [49]. The results obtained on the Pareto chart showed that MA and pH were significant at 95% confidence level. This means that the two independent variables played a significant role in the preconcentration of Sb(III). Therefore, further optimisation was required to give optimum conditions for MA and pH.

#### 3.2.1. Response Surface Methodology

**Figure 6:**
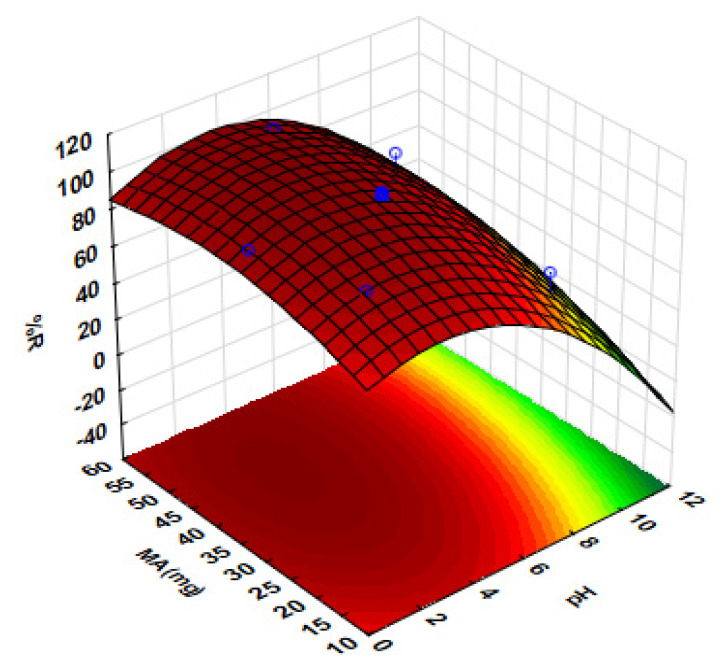
Response surface methodology for the preconcentration of Sb.

For further optimisation, response surface methodology (RSM) based on central composite design (CCD) was used to evaluate the interaction between MA and pH. The CCD matrix and respective analytical response are shown in Table S3. The 3D response surface plot shows the analytical response against individual factors (Figure 6). As can be seen in Figure 6, an enhanced analytical response (% recovery) at a pH value between 2 and 4 was observed and, for the MA, maximum recoveries were achieved at a mass between 50 and 60 mg. Above pH 4, lower recoveries were observed, and this might be due to the reduced interactions between the negatively charged analyte and positively charged adsorbent.

#### 3.2.2. Estimation of Optimum Conditions Using Desirability Functions

**Figure 7:**
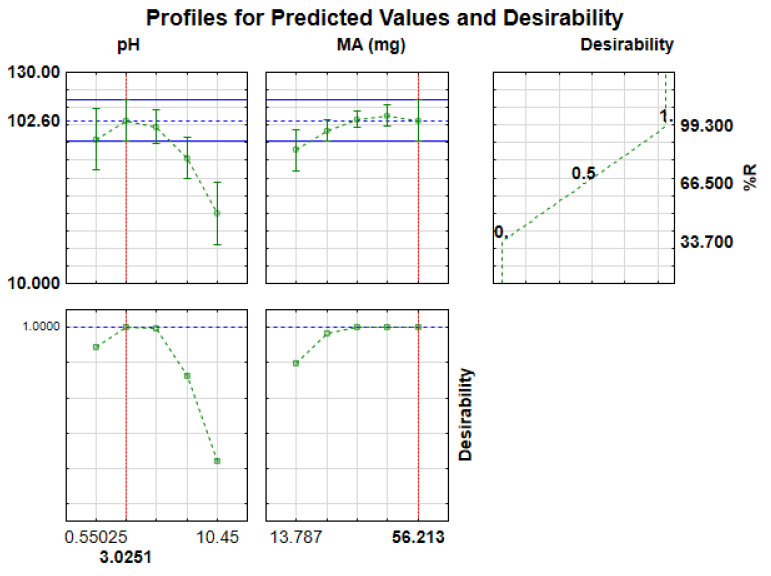
Desirability profile of predicted optimum conditions for the preconcentration of Sb(III).

In Figure 7, the desirability functions of 0.0, 0.5 and 1.0 were assigned to undesirable (33.7%), middle (66.5%) and desirable (maximum recoveries, (99.3%), respectively. Herein, the desirability score of 1.0 was chosen to estimate the desirable optimal parameters. Therefore, based on the screening results and desirability score of 1.0, the optimum conditions, were, 3.0, 56 mg, 3.0 mol L^−1^, 10 min, 20 min and 7.0 mL, for pH, MA, EC, ST, ET and EV, respectively. These conditions were confirmed experimentally (in triplicates) and the experimental recoveries (98.7 ± 1.2%) agreed with the RSM predicted value (99.3%) at 95% confidence level.

### 3.3. Scatchard Analysis, Adsorption Isotherms and Selectivity Studies

#### 3.3.1. Scatchard Analysis

**Figure 8:**
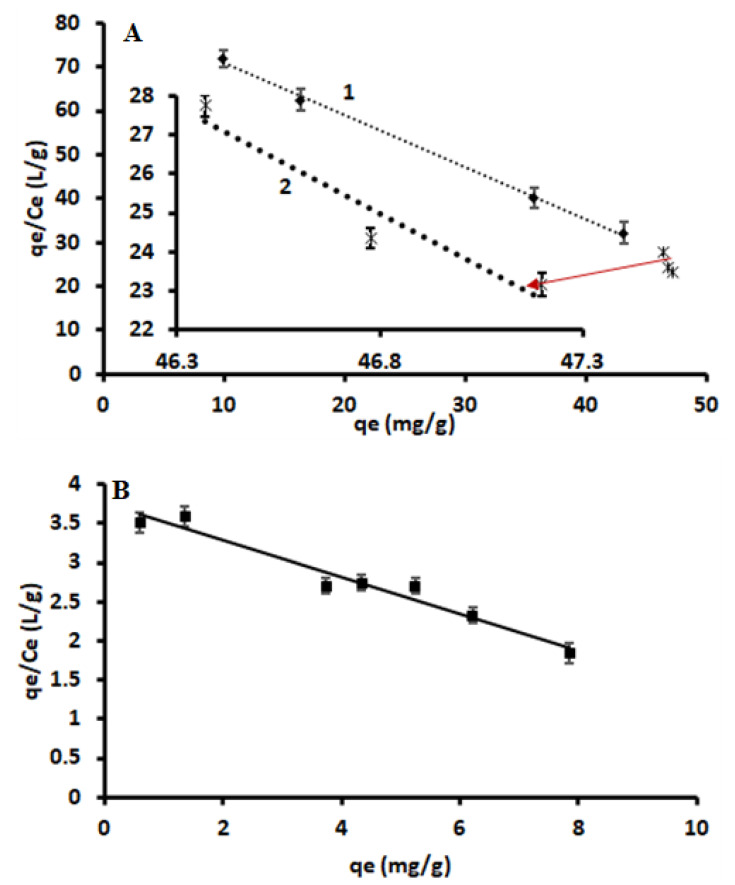
Scatchard plots of (**A**) Sb(III)-IIP-Fe_3_O_4_@SiO_2_@CNFs and (**B**) NIP-Fe_3_O_4_@SiO_2_@CNFs.

The theoretical maximum Sb(III) binding or adsorption capacity of IIP was also estimated using Scatchard plot obtained according to Equation (1).

(2) $$\frac{q_{e}}{C_{e}}=\frac{q_{max}-q_{e}}{K_{d}}$$

where *q_e_* (mg/g) is the adsorption or binding capacity at equilibrium, *C_e_* is the residual concentration of Sb(III) at equilibrium, *K_d_* (mg/L) is the equilibrium dissociation constant at binding sites and *q_max_* (mg/g) is the maximum binding [32]. The values of *K_d_* and *q_max_* were calculated from the slope and the intercept of the linear plot of *q_e_*/*C_e_* versus *q_e_* [32]. According to the literature, Scatchard plot shape is correlated to the nature of the interaction between the adsorbate and adsorbent [47,50]. For example, if the plot of *q_e_*/*C_e_* versus *q_e_* forms one straight line, this suggests that there is only one type of binding site on the surface of the sorbent [31]. Furthermore, when the Scatchard plot displays an anomaly from linearity (showing two linear plots in one set of data), the results suggest that the adsorbent has more than one type of binding site. These binding sites can be categorised as high-affinity (low *K_d_* value) and low-affinity (high *K_d_* value) binding sites [51,52]. Figure 8 shows the Scatchard plots of Sb(III)-IIP-Fe_3_O_4_@SiO_2_@CNFs and NIP-Fe_3_O_4_@SiO_2_@CNFs. As seen in Figure 8A, the Scatchard plot for Sb(III)-IIP-Fe_3_O_4_@SiO_2_@CNFs was nonlinear and it was divided into two linear sections that had two different slopes. As discussed earlier, these observations suggest that IIP@Fe_3_O_4_@CNF@SiO_2_ had two types of binding sites that have different affinities for the adsorption of Sb(III). On the contrary, the Scatchard plot of NIP-Fe_3_O_4_@SiO_2_@CNFs (Figure 8B) fitted to one linear curve, suggesting that NIP had only one type of binding site.

The slope and intercept of the fitted Scatchard plots were used to estimate the values maximum adsorption capacities and equilibrium dissociation constants for Sb(III)-IIP and NIP (Table 2). The *q_max_* and *K_d_* values from the higher affinity binding sites (Curve A-1) were found to be 0.162 13.4 mg/g and mg/L, respectively. In the low affinity binding sites (Curve A-2), the *K_d_* and *q_max_* values were 3.03 mg/L and 47.3 mg/g, respectively. Furthermore, the R^2^ of curves A-1 and A-2, confirmed existence of two types of binding sites in Sb(III)-IIP and lower R^2^ value (0.9215) for Curve A-2 suggested that the cavities in this region were not specific to Sb(III) adsorption. On the other hand, the *q_max_* and *K_d_* values for Sb(III)-IIP were 16.1 mg/g and 4.29 mg/L, respectively. As seen in Table 2, the highest dissociation equilibrium constant was obtained when NIP-Fe_3_O_4_@SiO_2_@CNFs was used as an absorbent. Overall, the trend of dissociation equilibrium constant in this study was as follows: K_dA-1_ < K_dA-2_ < K_dB_. These findings further demonstrate that IIP-Fe_3_O_4_@SiO_2_@CNFs had a higher affinity for Sb(III) than the NIP. Similar results have been reported in the literature [50,51,52].

#### 3.3.2. Adsorption Isotherms

**Figure 9:**
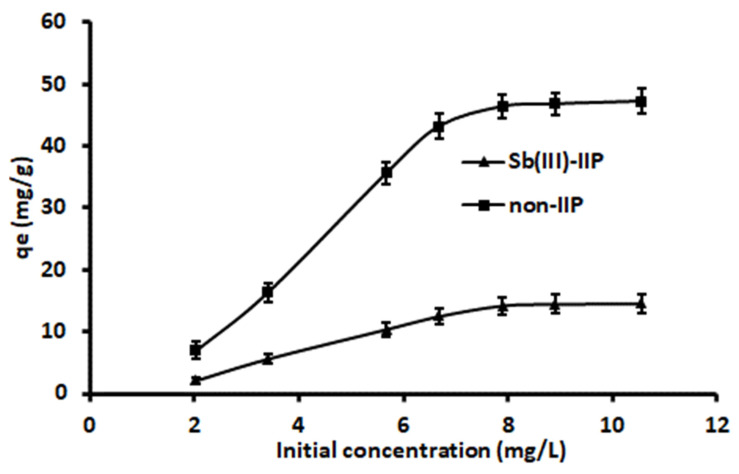
Equilibrium studies for Sb(III) adsorption onto the Sb(III)-IIP-Fe_3_O_4_@SiO_2_@CNFs and NIP-Fe_3_O_4_@SiO_2_@CNFs.

The adsorption of Sb(III) on IIP-Fe_3_O_4_@SiO_2_@CNFs and NIP-Fe_3_O_4_@SiO_2_@CNFs is shown in Figure 9. The Sb(III) adsorption or binding capacity of both IIP-Fe_3_O_4_@SiO_2_@CNFs and NIP-Fe_3_O_4_@SiO_2_@CNFs increased with increasing initial concentration until the saturation point was achieved. The adsorption capacity of IIP-Fe_3_O_4_@SiO_2_@CNFs was found to be higher than that of NIP-Fe_3_O_4_@SiO_2_@CNFs, suggesting that the imprinting played a significant role in the formatting of cavities that are specific to Sb(III). Isotherm models such as Langmuir and Freundlich were used to exam the adsorption behaviour of Sb(III)-IIP-Fe_3_O_4_@SiO_2_@CNFs and NIP-Fe_3_O_4_@SiO_2_@CNFs) towards Sb(III). The linear equations for the isotherm models and their constant parameters are presented in Table 3. The linear plots for Langmuir (*C_e_*/*q_e_* vs. *C_e_*) and Freundlich (ln *q_e_* vs. ln *C_e_*) models using IIP and NIP are shown in Figures S1–S4. The isotherm constant values were estimated from the slope and intercept of each plot (Table 3). The correlation of determination (R^2^) values was used to select the model that best explains adsorption results. As seen, the data for Sb(III)-IIP were best described by the Langmuir model (R^2^ = 0.9969) and the *q_max_* value was 47.4 mg g^−1^. Furthermore, the *q_max_* obtained from the Langmuir isotherm model was in agreement with the experimental adsorption capacity (46.7 mg g^−1^). These results suggest that the adsorption process was dominated by a monolayer sorption on homogenous adsorption or binding sites. The R^2^ (0.9806) of the Freundlich model revealed that there was relative correlation, but Langmuir model explains the adsorption data better. Similarly, adsorption data obtained using NIP-Fe_3_O_4_@SiO_2_@CNFs revealed that the Langmuir model (0.9912) fitted the data better than Freundlich model (0.9869). The adsorption capacity obtained in this study was higher than those reported by Zhang et al. [42] (39.6 mg g^−1^) and Shakerian et al. [44] (6.7 mg g^−1^) on the application of IIPs for adsorption antimony The imprinting factor (*α* = *q_max_* (IIP)/*q_max_* (NIP)) value was found to be 2.82, confirming successful imprinting.

#### 3.3.3. Selectivity

The selectivity studies of Sb(III)-IIP-Fe_3_O_4_@SiO_2_@CNFs and NIP-Fe_3_O_4_@SiO_2_@CNFs were carried out using a multielement sample solution containing Sb(III), Al(III), Cd(II), Cu(II), Sn(IV), Zn(II). Table 4 presents the selectivity experiment data and parameters, such as distribution ratio (D), adsorption capacities (*q_e_*), selectivity coefficient (*β*), relative selectivity coefficient (*β_r_*) and imprinting factor (*α*). Table 4 shows that the distribution ratio of Sb(III) ions for the Sb(III)-IIP adsorbent was 10 times higher than that of Sb(III)-NIP. On the contrary, the distribution ratio of co-exiting ions for Sb(III)-IIP was somehow lower than or equal to that of NIP except for Sn(IV). Furthermore, the adsorption capacity of Sb(III)-IIP towards Sb(III) was higher than other coexisting ions. Selectivity coefficient values of Sb(III)-IIP for Sb(III), Al(III), Cd(II), Cu(II), Sn(IV) and Zn(II) were 6–25 times higher than NIP, indicating that the IIP had higher binding specificity for Sb(III). Moreover, the imprinting factor value of the Sb(III) was greater than 1, demonstrating that the Sb(III)-IIP had a higher affinity toward the target analyte. These findings demonstrated that IIP-Fe_3_O_4_@SiO_2_@CNFs had strong binding property towards Sb(III) in the presence of other elements.

### 3.4. Analytical Performances

The linearity, limit of detection (LOD), limit of quantification (LOQ) and the precision (intra-day and inter-day) were used to evaluate the performance of the method. The linearity was assessed by analysing a series of standard solutions (0–150 µg L^−1^) using the UA-MSPE/ICP-OES method. The linear range was obtained between 0.44 and 100 µg L^−1^ with a correlation of determinations (R^2^) of 0.9976. The LOD and LOQ were 0.13 and 0.44 µg L^−1^, respectively. To investigate the precision of the method, a standard solution containing 100 µg L^−1^ Sb(III) ions was analysed repeatedly using the UA-MSPE/ICP-OES method. The intra-day (n = 10) and inter-day (n = 5 working days) expressed as relative standard deviations (RSD) were 2.4 and 4.7%, respectively. The enrichment factor of the proposed UA-DSPE/ICP-OES procedure was 71.3.

The analytical characteristics of the IIP-Fe_3_O_4_@SiO_2_@CNFs adsorbent were compared with those reported in the literature for the preconcentration of Sb(III) and are presented in Table 5. The developed method showed improved or similar analytical performance in terms of LOD, LOQ and linearity as compared to those reported by References [48,49,50,52]. However, the current method had high LOD and LOQ as well as narrow linear range as compared to those reported in the literature [2,17,39,51,53]. In addition, the preconcentration factor of the current method was lower than those reported elsewhere [2,17,48].

### 3.5. Application to Real Samples

The applicability of Sb(III)-IIP-Fe_3_O_4_@SiO_2_@CNFs adsorbent on selective extraction and preconcentration of Sb(III) in real water samples was studied. The method was applied for the analysis of Sb(III) in surface water collected from the local dams (DW1 and DW2) and river (RW). The concentrations of Sb(III) were 88.7, 9.7 and 40.5 μg L^−1^ in DW1, DW2 and RW, respectively. These results were compared with independent ICP-MS analysis, whereby the samples were analysed directly. The ICP-MS results and the ones obtained using the proposed method agreed at 95% confidence level. Therefore, it can be concluded that the use of Sb(III)-IIP-Fe_3_O_4_@SiO_2_@CNFs as an adsorbent in UA-MSPE resulted in the sensitivity and selectivity determination of Sb(III) in water samples. The concentrations of Sb(III) obtained in our study were compared with global concentrations of antimony in different water samples (Table 6). As seen, the levels of Sb(III) obtained in this study were comparable with those reported elsewhere [50,54,55]. However, they were found to be higher than those reported in Algeria, Mexico and Brazil (Table 6). High levels of Sb in surface water and ground water samples have been reported in China and Turkey (Table 6).

## 4. Conclusions

In this work, a simple, sensitive and highly selective method based on UA-MSPE/ICP-OES was developed for the analysis of trace amounts of Sb in surface water samples. Sb(III)-IIP-Fe_3_O_4_@SiO_2_@CNFs was used as an adsorbent in UA-MSPE. The SEM, XRD, TEM and EDS confirmed the successful synthesis of the nanocomposite. Parameters affecting the extraction and preconcentration of Sb(III) were optimised using RSM based on CCD. Under optimised conditions, the developed method displayed good analytical performance for extraction, preconcentration and determination of Sb(III) ions in environmental samples. The adsorbent displayed high adsorption capacity (47.8 mg g^−1^), low LOD (0.13 µg L^−1^) and high precision (2.4%) when compared to the previous study. Finally, the method was applied for analysis of Sb(III) in real surface water samples and the results agreed with the reference method. These results proved that Sb(III)-IIP-Fe_3_O_4_@SiO_2_@CNFs nanocomposite is the suitable adsorbent for trace analysis of Sb(III).

## Figures and Tables

**Table 1:** Lower and higher levels as well as central points of the investigated independent variables.

Parameters	Lower Level (−)	Central Point (0)	Higher Level (+)
Adsorbent Mass (mg)	20	35	50
Elution time (min)	5	17.5	30
Eluent volume (mL)	7	8.5	10
Eluent concentration (M)	1	3	5
Sonication time (min)	5	22.5	40
pH	2	5.5	9

**Table 2:** Scatchard plot parameters.

	Sb(III)-IIP	NIP
Regression equation	*q_e_*/*C_e_* = −6.1852*x* + 82.7 (Curve A-1)	*q_e_*/*C_e_* = −0.2333 + 3.7493
R^2^	0.9981	0.9557
*K_d_* (mg/L)	0.162	4.29
*q_max_* (mg/g)	13.4	16.1
Regression equation (Curve A-2)	*q_e_*/*C_e_* = −0.2282*x* + 15.04	
R^2^	0.9215	
*K_d_* (mg/L)	3.05	
*q_max_* (mg/g)	47.3	

**Table 3:** Adsorption isotherms models and constant *r* values.

Isotherms	Parameters	Sb(III)-IIP	Non-IIP
Langmuir	*q_max_* (mg/g)	47.4	16.8
$\frac{C_{e}}{q_{e}}=\frac{C_{e}}{q_{max}}+\frac{1}{q_{max}K_{L}}$	*K_L_*	1.81	0.29
	R^2^	0.9969	0.9912
Freundlich	*K_f_*	33.6	3.74
$lnq_{e}=lnK_{f}+\frac{1}{n}C_{e}$	*n*	1.73	1.4
	R^2^	0.9806	0.9869

**Table 4:** Selectivity studies.

Metal Ions	*q_e_* (mg/g)		Distribution Ratio (D, mL/g)		*β*		*β_r_*	*α*
	IIP	NIP	IIP	NIP	IIP	NIP		
Sb	46.1	16.6	22.2	2.19				2.78
Al	6.52	7.97	0.69	0.87	32.3	2.52	12.8	0.82
Cd	8.41	8.53	0.92	0.94	24.1	2.33	10.3	0.99
Cu	3.70	8.46	0.37	0.93	60.0	2.35	25.5	0.44
Sn	11.2	12.1	2.19	1.44	10.2	1.52	6.67	0.93
Zn	6.24	7.47	0.66	0.80	33.9	2.71	12.5	0.83

**Table 5:** Comparison of the proposed adsorbent with other reported adsorbent for extraction and preconcentration of Sb.

Analyte	Adsorbent	Linear Range (μg L^−1^)	LOD (μg L^−1^)	LOQ (μg L^−1^)	PF	RSD (%)	Refs
Sb	PAN	0.027–650	0.008	0.027	150	1.8–4.1	[17]
Sb(III)	SiO_2_/Al_2_O_3_/SnO_2_	0.50–5.00	0.17	0.56	136	-	[53]
Sb(III)	TAR	0.5–180	0.13	0.43	-	0.9	[54]
Sb	Zr-NPs	30–250	8.0	26.8	-	-	[55]
Sb(III)	IIP	-	0.04	0.13	-	2.3	[42]
Sb, Sb(III)	Mercapto-functionalised hybrid sorbent	-	0.0025	0.008	-	1.6	[56]
Sb(III)	TAC	0.93–180	0.28	0.93	-	3.6	[54]
Sb	IIP	-	0.0039	0.13	-	3.1	[44]
Sb	DBD	1–200	0.2	0.67	-	3	[57]
Sb(III)	POIP	-	0.006	0.02	100	4.2	[2]
Sb	PIL	0.20–200	0.084	0.28	-	<9	[58]
Sb(III)	IIP-Fe_3_O_4_@SiO_2_@CNFs	0.44–100	0.13	0.44	71.3	2.4 and 4.7	This work

IIP: ion imprinted polymers, PIL: polymeric ionic liquid, DBD: dielectric barrier discharge, POIP: polystyrene oleic acid imidazole polymer, TAR: 4-(2-thiazolylazo) resorcinol, TAC: 2-(2-thiazolylazo)-p-cresol, PAN: peroxyacetyl nitrate, Zr-NPs: zirconium nanoparticles, SiO_2_/Al_2_O_3_/SnO_2_: silicon dioxide/aluminium oxide/tin oxide.

**Table 6:** Global concentration of Sb in water samples.

Country	Matrix	Concentration of Sb (μg L^−1^)	Refs
Mexico	Drinking water	0.28–2.30	[12]
China	Ground water	6–30,000	[59]
Algeria	Drinking water	0.50–1.12	[60]
Pakistan	Drinking water	28	[61]
Brazil	Mineral and surface water	0.26–0.30 and 0.41–1.23	[62]
Brazil	Mineral water	0.54–1.04	[53]
Turkey	Wastewater	300–2000	[63]
China	Surface water	30–150	[64]
Greece	Tap water	10–100	[65]
China	Wastewater	330–11,400	[66]
South Africa	Dam and river water	9.7–88.7	This work

## Data Availability

Data are included as the Supplementary Materials and if raw data are required, they will be made available upon request.

## Supplementary Materials

The following are available online at https://www.mdpi.com/article/10.3390/polym14010021/s1, Table S1: Operating parameters of an ICP-OES Table S2: Fractional factorial design matrix and analytical response, Table S3: Central composite design matrix and analytical response, Figure S1: Linearised Langmuir isotherm model for IIP, Figure S2: Linearised Freundlich isotherm model for IIP, Figure S3: Linearised Langmuir isotherm model for NIP, Figure S4: Linearised Freundlich isotherm model for NIP.
