# Supplementary material for: Magnetic Ion Imprinted Polymers (MIIPs) for Selective Extraction and Preconcentration of Sb(III) from Environmental Matrices
Source: Polymers (Basel). 2021 Dec 22;14(1):21. doi: 10.3390/polym14010021 (PMC8747241; doi:10.3390/polym14010021)
Supplement: Supplementary file 1 [file polymers-14-00021-s001.zip › polymers-1514117-supplementary.pdf]

Magnetic ion imprinted polymers (MIIPs) for selective extraction and preconcentration of Sb(III) from environmental matrices

Silindokuhle Jakavula[a,b], Nkositetile. Raphael Biata[a,b], Kgogobi M. Dimpe[a], Vusumzi E. Pakade[c], Philiswa Nosizo Nomngongo[a,b*]

[a]*Department of Chemical Sciences, University of Johannesburg, Doornfontein Campus, P.O. Box 17011, Johannesburg, 2028, South Africa*

[b]*Department of Science and Innovation-National Research Foundation South African Research Chair Initiative (DSI-NRF SARChI): Nanotechnology for Water, University of Johannesburg, Doornfontein, 2028, South Africa*

[c]*Department of Chemistry, Vaal University of Technology, Private Bag X 021, Vanderbijlpark, South Africa.*

Supplementary information

Instrumentation

Inductively coupled plasma-optical emission spectrometer (ICP-OES) (iCAP 6500 Duo, Thermo Scientific, UK) equipped with a charge injection device (CID) detector was used in the determination metal ions 'concentrations. The samples were introduced with a concentric nebulizer and a cyclonic spray chamber. The operating parameters of the instrument are presented in Table 1. The most prominent atomic and ionic analytical spectral wavelength line of the selected element for the investigation was Sb 206.833 nm. Branson 5800 Ultrasonic Cleaner (UK) and Eppendorf 5702 Centrifuge (Germany) were used for ultrasonic-assisted extraction and centrifugation, respectively.

Table S1: Operating parameters of an ICP-OES

ICP-OES parameters	Conditions
RF generator power/W	1150
Frequency of RF generator/MHz	40
Coolant gas flow rate/ L min^{-1}	12

[*]*Corresponding author: E-mail address*: pnnomngongo@uj.ac.za or nomngongo@yahoo.com; Tel:+27115596187

Carrier gas flow rate/ L min^{-1}	0.7
Auxiliary gas/ L min^{-1}	1.0
Max integration times/s	15
Pump rate/rpm	50
Viewing configuration/Touch mode	Axial
Replicate	3
Flush time/s	30

Preparation of Sb(III)-Ion imprinted polymer modified with Fe_3O_4@CNFs@SiO_2

The synthesis of Sb(III)-IIP was done according to [39], where the complex of Sb(III) with ammonium pyrrolidine dithiocarbamate (APDC) was prepared by stirring 4 mmol of APDC dissolved in 10 mL of 2-methoxy ethanol containing 1 mmol of antimony (III) for 20 min. Then the precipitated complex was filtered, washed with 2-methoxy ethanol and was air dried. The yellow complex was dissolved in a 15 mL mixture of chloroform and ethanol (2:1) solvents and 150 mg of Fe_3O_4@CNFs@SiO_2 was added. It was then mixed with styrene as the monomer (8 mmol) and ethylene glycol dimethacrylate (EGDMA) as the crosslinker (32 mmol) in the presence of 50 mg of AIBN as the initiator. The polymerization mixture was cooled to 0 ºC, purged with N_2 for 10 min, sealed and thermally polymerized in a water bath. The temperature was then slowly raised from room temperature to 55 ºC and was maintained at 55 ºC for 24 h. The resulting polymeric material was washed thoroughly with the mixture of deionized water and ethanol, dried, ground and sieved to obtain antimony ion imprinted polymer particles. The Sb(III) ions were removed from the synthesized polymeric material upon treatment with 400 mL of HCl (50% v/v) for 6 h. The removal process was continued until there was no detection of antimony. The polymer particles were then washed thoroughly with water and dried in an oven at 60 ºC for further use.

Supplementary data

Table S2: Fractional factorial design matrix and analytical response

Runs	ST(min)	MA(mg)	pH	EC (mol/L)	EV(mL)	ET(min)	%R (Sb)
1	5.0	20.0	2.0	1.0	7.0	5.0	10.3
2	40.0	20.0	2.0	1.0	10.0	5.0	6.5
3	5.0	50.0	2.0	1.0	10.0	20.0	35.9
4	40.0	50.0	2.0	1.0	7.0	20.0	15.3
5	5.0	20.0	9.0	1.0	10.0	20.0	10.4
6	40.0	20.0	9.0	1.0	7.0	20.0	11.1
7	5.0	50.0	9.0	1.0	7.0	5.0	57.9
8	40.0	50.0	9.0	1.0	10.0	5.0	59.6
9	5.0	20.0	2.0	5.0	7.0	20.0	5.8
10	40.0	20.0	2.0	5.0	10.0	20.0	13.9
11	5.0	50.0	2.0	5.0	10.0	5.0	15.1
12	40.0	50.0	2.0	5.0	7.0	5.0	15.3
13	5.0	20.0	9.0	5.0	10.0	5.0	13.4
14	40.0	20.0	9.0	5.0	7.0	5.0	13.2
15	5.0	50.0	9.0	5.0	7.0	20.0	66.0
16	40.0	50.0	9.0	5.0	10.0	20.0	63.3
17 (C)	22.5	35.0	5.5	3.0	8.5	12.5	16.6
18 (C)	22.5	35.0	5.5	3.0	8.5	12.5	9.9
19 (C)	22.5	35.0	5.5	3.0	8.5	12.5	9.8

Table S3: Central composite design matrix and analytical response

Runs	pH	MA	%R (Sb)
**1**	2.0	20.0	93.3
**2**	2.0	50.0	99.3
**3**	9.0	20.0	60.7
**4**	9.0	50.0	76.0
**5**	0.55	35.0	97.7
**6**	10.5	35.0	33.7
**7**	5.5	13.8	70.8
**8**	5.5	56.2	98.7
**9 (C)**	5.5	35.0	97.1
**10 (C)**	5.5	35.0	97.7
**11 (C)**	5.5	35.0	96.6
**12 (C)**	5.5	35.0	97.3
**13 (C)**	5.5	35.0	98.8
**14 (C)**	5.5	35.0	97.7
**15 (C)**	5.5	35.0	98.0
**16 (C)**	5.5	35.0	96.5

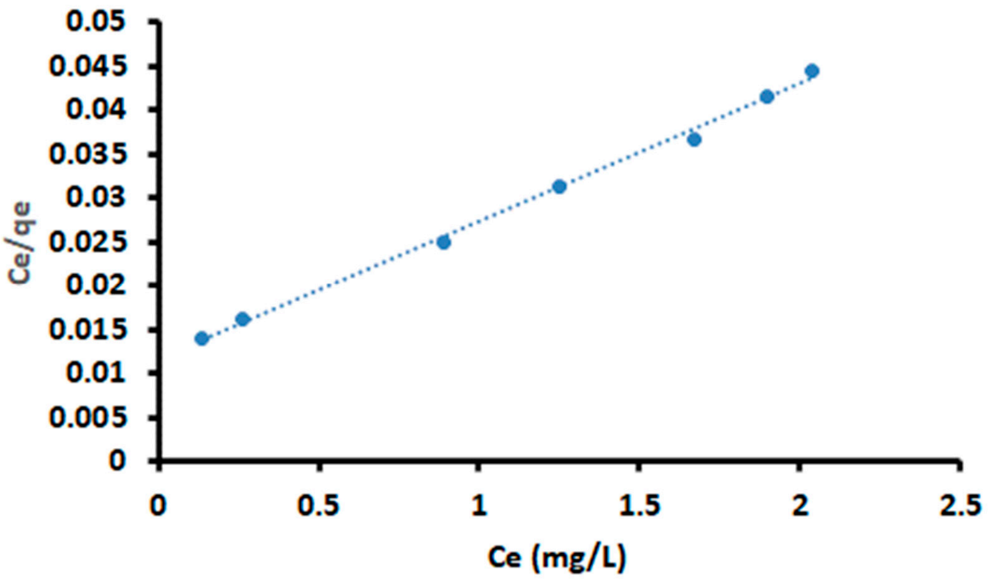

Figure S1: Linearised Langmuir isotherm model for IIP

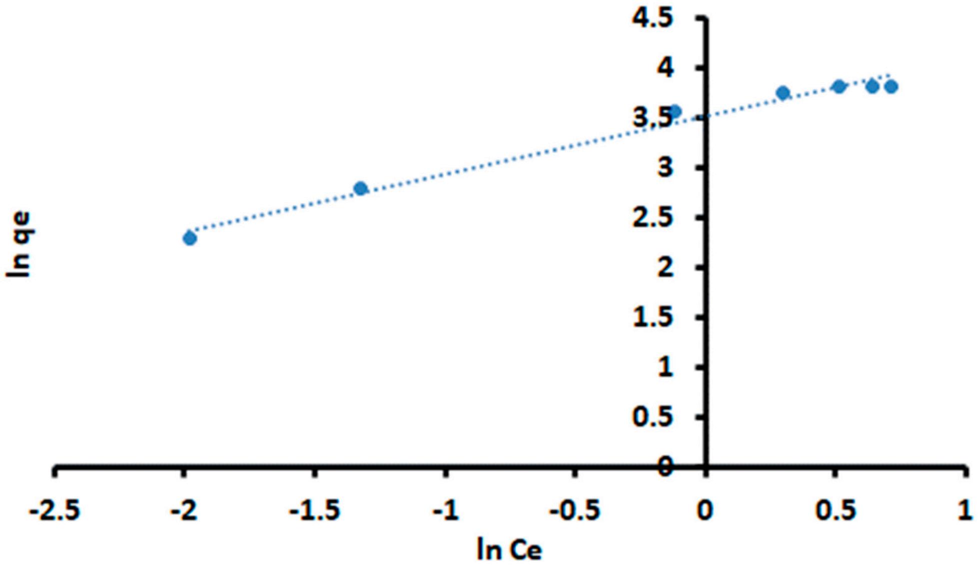

Figure S1: Linearised Freundlich isotherm model for IIP

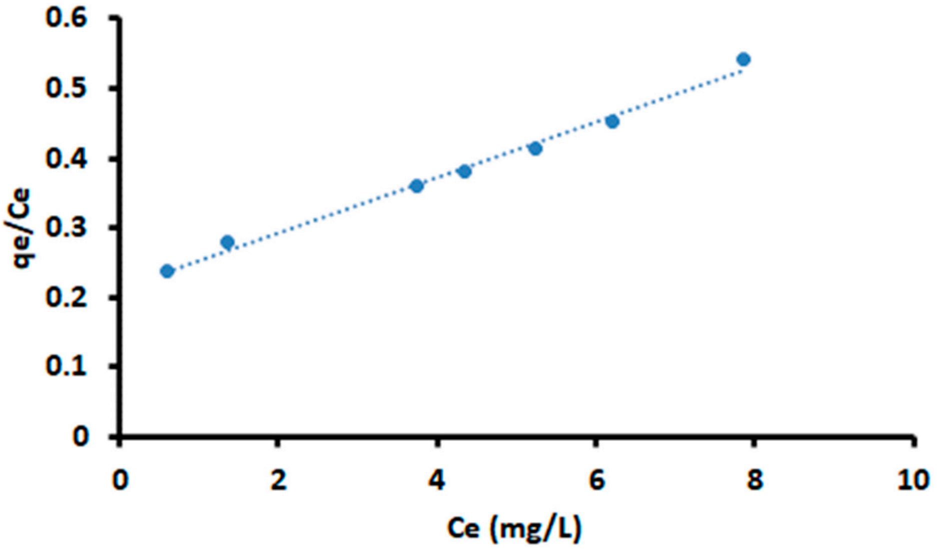

Figure S2: Linearised Langmuir isotherm model for NIP

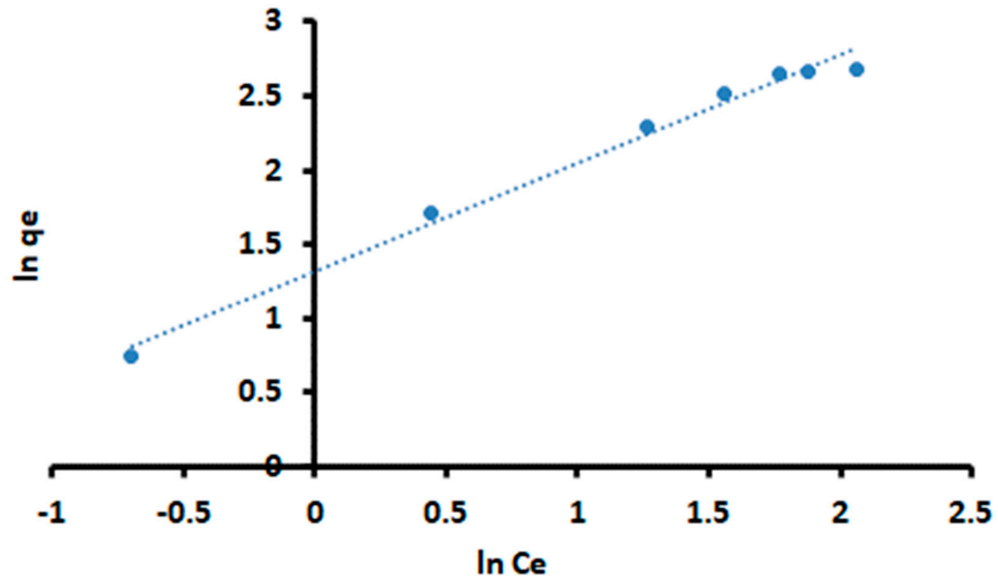

Figure S3: Linearised Freundlich isotherm model for NIP
